# The detrimental effect of autoimmune hepatitis in pregnancy: a systematic review and meta-analysis

**DOI:** 10.1097/MS9.0000000000003905

**Published:** 2025-09-16

**Authors:** Hamza A. Khan, Mahnoor Khattak, Maaz B. Badshah, Nayab Sami, Subtain Hassan, Qaisar A. Khan, Sumaira Iram, Muhammad Mudasser, Farman Ullah, Dipta A. Biswas

**Affiliations:** a Khyber Teaching Hospital, MTI KTH Peshawar, Peshawar, Pakistan; b Shifa International Hospital, Islamabad, Pakistan; c Dhaka Medical College, Dhaka, Bangladesh

**Keywords:** autoimmune hepatitis, gestation, hepatitis, liver, pregnancy

## Abstract

**Background:**

Autoimmune hepatitis (AIH), a chronic inflammatory liver disease, poses unique challenges during pregnancy. In AIH, the immune response triggers inflammation, potentially leading to cirrhosis if left untreated. The study aims to provide comprehensive insights into the relationship between AIH and pregnancy, guiding clinicians in optimal maternal management.

**Methods:**

Our meta-analysis includes data searched up to 1 January 2024. Eligibility criteria encompassed studies focusing on pregnant individuals with AIH, excluding case reports, letters, reviews, and incomplete papers. Data extraction, quality assessment using Cochrane risk of bias or Newcastle–Ottawa Scale, and quantitative analysis through Comprehensive Meta-Analysis were performed.

**Results:**

The literature search included 15 studies, involving 38 679 pregnant women with AIH across 10 countries. Maternal outcomes, such as disease flare [event rate (*E*) = 0.228, 95% CI: 0.13–0.36; *P* = 0.000] and maternal deaths, indicated significant associations. The incidence of gestational diabetes was higher in AIH patients (*E* = 0.091, 95% CI: 0.043–0.184; *P* = 0.001), showing notable heterogeneity. Fetal outcomes demonstrated significant associations with preterm births (*E* = 0.190, 95% CI: 0.131–0.268; *P* = 0.000), fetal loss (*E* = 0.142, 95% CI: 0.082–0.236; *P* = 0.000), and congenital anomalies (*E* = 0.041, 95% CI: 0.026–0.065; *P* = 0.000).

**Conclusion:**

This systematic review contributes a comprehensive overview of the complex interplay between AIH and pregnancy, providing evidence for heightened risks and implications on maternal and fetal outcomes. Clinicians can leverage these findings for informed decision-making, while the study enriches the broader field of autoimmune and reproductive health.

## Introduction

First discovered in 1951, autoimmune hepatitis (AIH) is a chronic liver disease characterized by autoimmune or self-mediated inflammation, leading to progressive hepatocellular damage^[1]^. Despite being present in all age groups and demographics, it has been observed that women are primarily affected by this disorder. The male-to-female ratio is commonly quoted as 1:6. The global incidence and prevalence of AIH were determined to be 1.28 cases per 100 000 inhabitant-years^[2,3]^. Although AIH is a chronic disease that can progress to cirrhosis, hepatocellular carcinoma, liver transplantation, and/or death, it can often start with an episode of acute hepatitis hence making early diagnosis challenging^[1]^.

**HIGHLIGHTS**

- Pregnancy is associated with higher risk of maternal and fetal complications in people with AIH.
- The heightened risks identified, including increased disease flare, maternal mortality, GDM, preterm births, fetal loss, and congenital anomalies, highlight the importance of vigilant clinical management for pregnant individuals with AIH.
- Clinicians should consider these findings in the context of individual patient characteristics, emphasizing personalized care and tailored interventions.

According to research, the development of AIH is influenced by both genetic predisposition and environmental causes. Genetic investigations have revealed that variations of the human leukocyte antigen contribute to an increased risk of developing AIH. More recently, environmental variables (such as viral infections) have been linked to the development of AIH. In patients with a higher genetic vulnerability to AIH, immune responses to liver autoantigens may be initiated via molecular mimicry, in which immune responses to exogenous pathogens are directed toward structurally identical self-proteins^[4-6]^.

The clinical presentation of adults with AIH varies greatly. Most individuals show no signs or symptoms of hepatobiliary illness but have elevated serum aspartate transaminase and alanine transaminase levels. Nonspecific, moderate fatigue is prevalent among these otherwise asymptomatic people. Extrahepatic autoimmune disorders frequently cause indications or symptoms in patients.

The treatment of AIH is centered around immunosuppression to halt the autoimmune attack on the liver. Corticosteroids, particularly prednisone, are the cornerstone of therapy, often administered alongside azathioprine for a synergistic effect. This combination aims to induce and maintain remission, preventing disease progression and complications. While effective, the long-term use of corticosteroids poses concerns related to side effects, necessitating careful monitoring. For patients with contraindications or intolerances to standard therapy, alternative immunosuppressive agents, such as mycophenolate mofetil or tacrolimus, may be considered. A crucial aspect of AIH management is achieving a delicate balance between immunosuppression and overtreatment. Thus, regular monitoring of liver function, autoantibodies, and side effects is imperative to tailor treatment strategies to individual patient needs, ensuring optimal outcomes in the chronic management of AIH^[7-11]^.

While the disorder has a better prognosis in the general population due to rapidly expanding therapy options, data on pregnancy-related AIH is still limited. Previous investigation has demonstrated that autoimmune illnesses increase the possibility of obstetric difficulties, as well as neonatal morbidity and mortality^[3,12]^. Pregnancy, a physiological state characterized by dynamic changes, introduces additional layers of complexity when AIH is in the picture. Addressing the evolving disease activity during pregnancy is crucial for optimal maternal management. Moreover, exploring the impact on diabetes mellitus incidence becomes paramount, considering the intricate interplay between autoimmune conditions and metabolic health. The heightened likelihood of cesarean section in AIH pregnancies adds an intriguing dimension, warranting examination. Unraveling the reasons behind this increased propensity provides insights into the intersection between autoimmune liver conditions and obstetric outcomes^[13,14]^.

Complications such as variceal bleeding, while inherent to AIH, carry particular significance in the context of pregnancy. Understanding the risk and implications of such events is crucial for anticipating and managing these potentially life-threatening scenarios. Hence, this systematic review aims to synthesize existing knowledge, providing a comprehensive overview of the intricate relationship between AIH and pregnancy.

## Material and methods

Our present meta-analysis was performed according to the guidelines of Preferred Reporting Items for Systematic Reviews (PRISMA) and Assessing the Methodological Quality of Systematic Reviews (AMSTAR-2) guidelines^[15,16]^.

### Search strategy

The literature search employed a systematic approach on PubMed, Scopus, and Web of Science. Data were searched from inception till 1 January 2024 without any language or geographical restrictions, ensuring a comprehensive overview. We also explored gray literature via conference proceedings, dissertations, bibliographies, and other relevant organizational websites to capture unpublished or non-peer-reviewed data. The following search terms were employed: “pregnant,” “pregnancy,” “gravidity,” “gestation,” “autoimmune,” “hepatitis,” “AIH,” and “AILD.”

### Eligibility criteria

- Age range: 18–45 years;
- Gestational age: any trimester;
- AIH definition: clinical or biopsy-confirmed.

The inclusion criteria of our study were defined specifically addressing pregnant individuals with AIH. This comprehensive approach encompassed both observational studies and clinical trials to provide a well-rounded perspective. Our chosen outcomes were categorized into maternal and fetal domains, incorporating outcomes such as maternal mortality, gestational diabetes mellitus (GDM), and disease flare-up (based on transaminase levels exceeding 2× the upper normal limit). On the fetal side, our focus extended to outcomes like demise (any pregnancy loss after viability threshold, i.e., 24 weeks), congenital abnormality, and preterm birth. We eliminated case reports, letters, reviews, and incomplete papers to ensure the robustness and reliability of the selected studies.

### Data extraction method

Data extraction was employed via a premade form, capturing study characteristics, participant demographics, and relevant outcomes. All titles and abstracts were screened for inclusion according to the set criteria. Full texts of selected articles were screened for in-depth review by two investigators, and data were extracted from eligible articles into a pre-structured Microsoft Excel data sheet (Version 2019, Microsoft). Disagreements were resolved by consultation with another author.

### Quality assessment

The Cochrane risk of bias assessment tool or the Newcastle–Ottawa Scale (NOS) was used for quality assessment.

### Quantitative assessment

Comprehensive Meta-Analysis (3.0) was employed for data analysis. This quantitative approach aimed to synthesize findings, providing a robust understanding of the impact of AIH on pregnancy outcomes. The heterogeneity of the data was evaluated using the *I*-squared (*I*^2^) statistic to quantify variability across studies. An *I*^2^ value above 50% was considered indicative of substantial heterogeneity. For meta-analysis, a random-effects model was employed to account for potential variations across studies, considering differences in study design, population characteristics, and methodologies. This model was chosen due to the observed heterogeneity in the included studies, ensuring a more generalized interpretation of the pooled results

## Results

### Literature search

**Figure 1.**
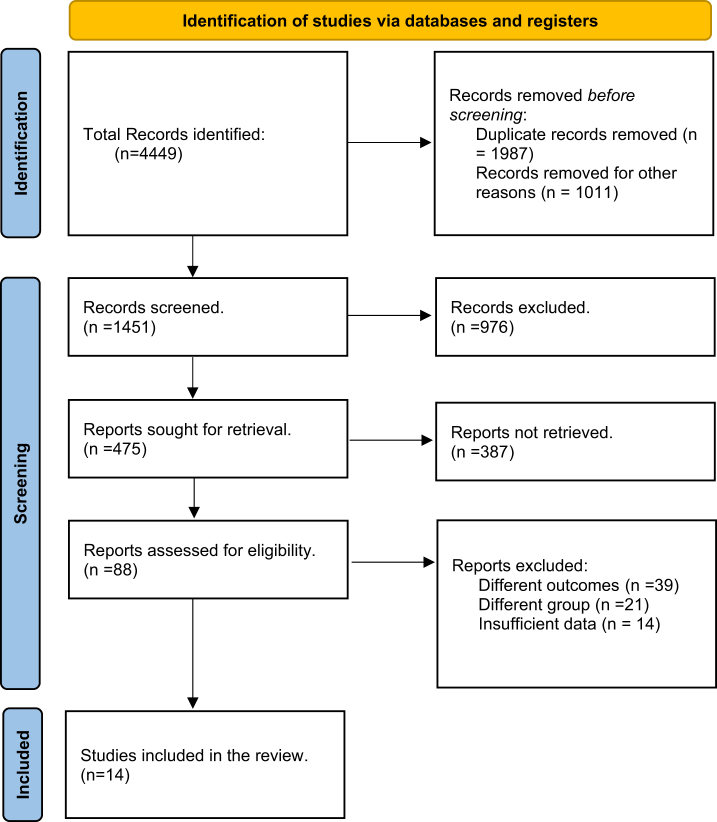
Preferred Reporting Items for Systematic Reviews and Meta-Analyses (PRISMA) flow chart of literature search.

A total of 4449 articles were retrieved. After the removal of duplicates and other articles, 1451 articles were screened via titles and abstracts. Of these, 475 articles were sought for retrieval, and finally, 88 articles were selected for in-depth review. Fourteen observational studies were included in the final qualitative and quantitative meta-analysis^[3,14,17–28]^. This selection process is illustrated in the PRISMA flowchart (Fig. 1).

### Risk of bias assessment

The included studies exhibited a mixed risk of bias across various domains. Overall, the studies showed a great effort in reporting and minimizing bias. Notably, studies by Gronbaek *et al*., Sharma *et al.*, and Kilani *et al.* demonstrated commendable transparency by reporting on all eight bias domains^[24,25,27]^.

Among the studies, Groebaek *et al.* (Risk of Bias/ROB score of 7/8) and Sharma *et al.* (ROB score of 6/8) emerged with the lowest risk of bias^[24,27]^. These studies were both prospective cohort studies with well-defined inclusion and exclusion criteria, adequate methods for randomization and blinding, and low attrition rates. Kilani *et al.* (ROB score of 5/8) and Nomuras (ROB score of 4/8) also had relatively low risk of bias^[19,25]^. These studies were both retrospective cohort studies with well-defined inclusion and exclusion criteria, but they had higher attrition rates than the Groebaek *et al.* and Sharma *et al.* studies.

However, concerns regarding reporting bias were generally low, as most studies adhered to guidelines and presented balanced outcomes. Table 1 presents detailed risk of bias.

**Table 1:** Risk of bias for included studies

	Selection				Comparability	Outcome			
Study	1	2	3	4	5	6	7	8	Total
Izumi *et al*., 2003^[26]^	_	_	☆	_	_	☆	☆	☆	4
Schramm *et al*., 2006^[17]^	☆	_	_	☆	_	_	☆	☆	4
Terrabuio *et al*., 2009^[18]^	☆	_	☆	☆	_	☆	☆	_	5
Nomuras *et al*., 2013^[19]^	_	_	☆	_	_	☆	☆	_	3
Westbrook *et al*., 2012^[3]^	☆	☆	☆	☆	☆	☆	☆	☆	8
Braga *et al*., 2016^[14]^	☆	_	_	_	_	☆	_	_	2
Llovet *et al*., 2019^[20]^	☆	☆	☆	_	☆	☆	☆	_	6
Olsen *et al*., 2021^[28]^	☆	-	☆	-	-	☆	☆	☆	5
Stokkeland., *et al* 2016^[21]^	☆	☆	☆	☆	☆	☆	_	☆	7
Gronbaek *et al*., 2018^[27]^	☆	☆	☆	☆	☆	☆	☆	☆	8
Borssén *et al*., 2015^[23]^	☆	-	☆	-	-	☆	☆	☆	5
Wang *et al*., 2023^[22]^	☆	☆	☆	_	☆	_	☆	☆	6
Sharma *et al*., 2023^[24]^	☆	☆	☆	☆	☆☆	☆	☆	☆	9
Kilani *et al*., 2023^[25]^	☆	☆	☆	☆	☆☆	☆	☆	☆	9

☆: Study meets the criteria for low risk of bias- : Study does not meet the criteria for low risk of bias

### Study characteristics

This meta-analysis synthesized findings from 14 studies exploring the link between AIH and pregnancy^[3,14,17–28]^. The studies spanned 10 countries and involved data from a total of 38 679 pregnant women with AIH. The average age of mothers across the studies was 30.6 years. Notably, five studies utilized a prospective cohort design, while the remaining 10 studies were retrospective. A detailed tabulation of baseline characters is presented as Table 2. Outcomes of each study are provided in Table 3.

**Table 2:** Baseline characteristics of included studies

Author, year	Country	Type of study	No. of patients	Total pregnancies	Age	Cirrhosis
Izumi *et al*., 2003^[26]^	Japan	Prospective Cohort	10	11	32 (26–37)	0/10
Schramm *et al*., 2006^[17]^	Germany	Retrospective	22	42	29 (17–37)	4/22
Terrabuio *et al*., 2009^[18]^	Brazil	Retrospective	39	51	24 (17–34)	26/39
Nomauras *et al*., 2012^[19]^	Brazil	Retrospective	10	12	25 (16–33)	5/10
Westbrook *et al*., 2012^[3]^	UK	Prospective Cohort	53	81	26 (16–42)	21/53
Brossen *et al*., 2015^[23]^	Sweden	Prospective Cohort	58	100	29 (24-40)	23/58
Braga *et al*., 2016^[14]^	Portugal	Retrospective	7	9	31.3 (25–43)	2/7
Stokkeland *et al*., 2016^[21]^	Sweden	Prospective	171	171	NA	
Grønbaek *et al*., 2018^[27]^	Denmark	Retrospective	70	70	NA	29/70
Llovet *et al*, 2019^[20]^	Italy	Retrospective	36	46	33 (29–34)	7/36
Olsen *et al*., 2021^[28]^	UK	Retrospective	20	27	29 (24–35)	12/27
Wang *et al*., 2023^[22]^		Retrospective	60	60	29.6	10/60
Kilani *et al*., 2023^[25]^	USA	Retrospective Cohort	1095	1095	NA	285/1095
Sharma *et al*., 2023^[24]^	Sweden	Retrospective Cohort	202	306	31.2 (28.3-34.7)	36/202

**Table 3:** Outcomes of each study

Author, year	Maternal outcomes			Fetal outcomes		
	Flare	Maternal death	Gestational diabetes	Pre-term	Fetal loss	Congenital abnormality
Izumi *et al*., 2003^[26]^	0/10	0/10	NA	0/9	1/10	0/9
Schramm *et al*., 2006^[17]^	9/42	1/42	NA	17/35	7/42	1/35
Terrabuio *et al*., 2009^[18]^	14/51	0/51	NA	6/38	13/54	2/38
Nomauras *et al*., 2012^[19]^	1/12	0/12	NA	5/12	1/13	0/12
Westbrook *et al*., 2012^[3]^	6/53	4/53	NA	12/61	20/81	1/61
Brossen *et al*., 2015^[23]^	3/58	NA	NA	22/100	31/100	1/69
Braga *et al*., 2016^[14]^	2/9	0/9	NA	2/6	3/9	0/6
Stokkeland *et al*., 2016^[21]^	NA	NA	8/171	14/171	NA	6/171
Grønbaek *et al*., 2018^[27]^	NA	NA	NA	12/70	0/70	5/70
Llovet *et al*, 2019^[20]^	10/36	0/36	NA	1/44	2/46	0/44
Olsen *et al*., 2021^[28]^	15/20	NA	3/20	8/27	NA	NA
Wang *et al*., 2023^[22]^	NA	NA	15/60	15/50	10/60	NA
Kilani *et al*., 2023^[25]^	NA	NA	140/1095	90/1095	NA	NA
Sharma *et al*., 2023^[24]^	NA	NA	7/202	51/303	3/303	2/306

NA: not available.

### Publication bias

**Figure 2.**
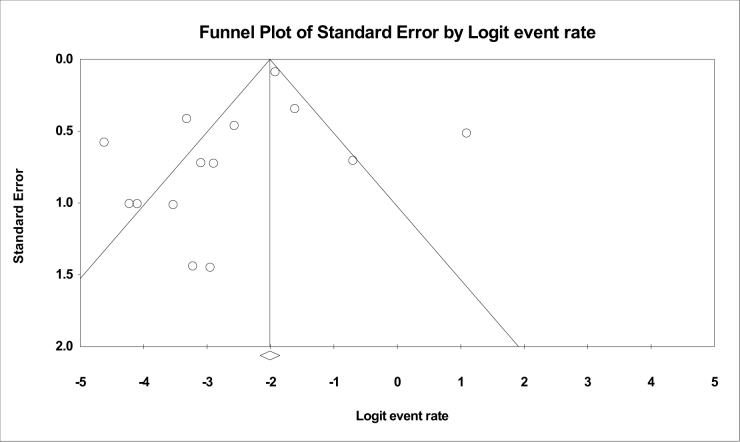
Publication bias.

The publication bias between studies is illustrated in Figure 2.

### Quantitative analysis

#### Maternal outcomes

##### Disease flare

**Figure 3.**
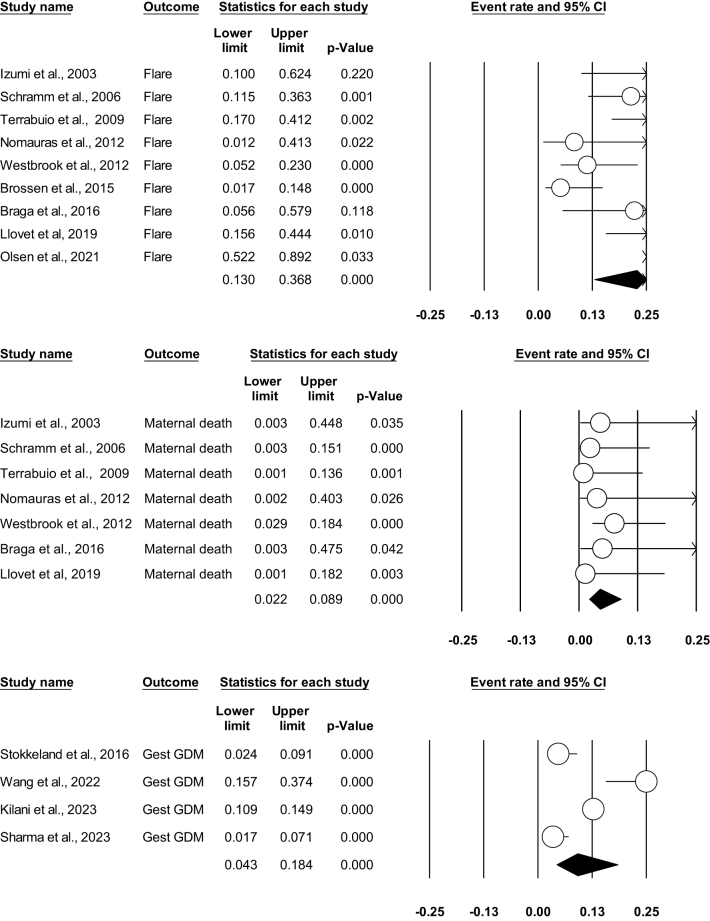
(A) Forest plot showing flare of disease. (B) Forest plot showing maternal death. (C) Forest plot showing gestational diabetes mellitus (GDM).

The event rate for maternal disease flare was found to be 0.228 (95% CI: 0.13–0.36; *P* = 0.000) across nine studies^[3,14,17–20,23,26,28]^. The *Z*-score of −3.519 and a significant *P*-value of 0.000 indicate a substantial association, highlighting the heightened risk of disease exacerbation during pregnancy. A moderate level of heterogeneity among the included studies were found (*I*^2^ = 76.838; *P* = 0.000). Figure 3A illustrates the forest plot for disease flare.

##### Maternal deaths

A total of seven out of 15 studies reported maternal mortality^[3,14,17–20,26]^. Our analysis found that the women with AIH had statistically significant maternal mortality (*E* = 0.045; 95% CI: 0.022–0.089; *P* = 0.000) between the groups. No heterogeneity was found between studies in this analysis (*I*^2^ = 0%; *P* = 0.738) (Fig. 3B).

##### Gestational diabetes

Only four studies reported GDM as one of their outcomes^[21,22,24,25]^. A significant statistical relationship was found, and our analysis revealed that AIH patients had a higher GDM rate (*E* = 0.091; 95% CI: 0.043–0.184; *P* = 0.001). High heterogeneity was found between our studies (*I*^2^ = 89.9; *P* = 0.000) (Fig. 3C).

#### Fetal outcomes

##### Preterm births

**Figure 4.**
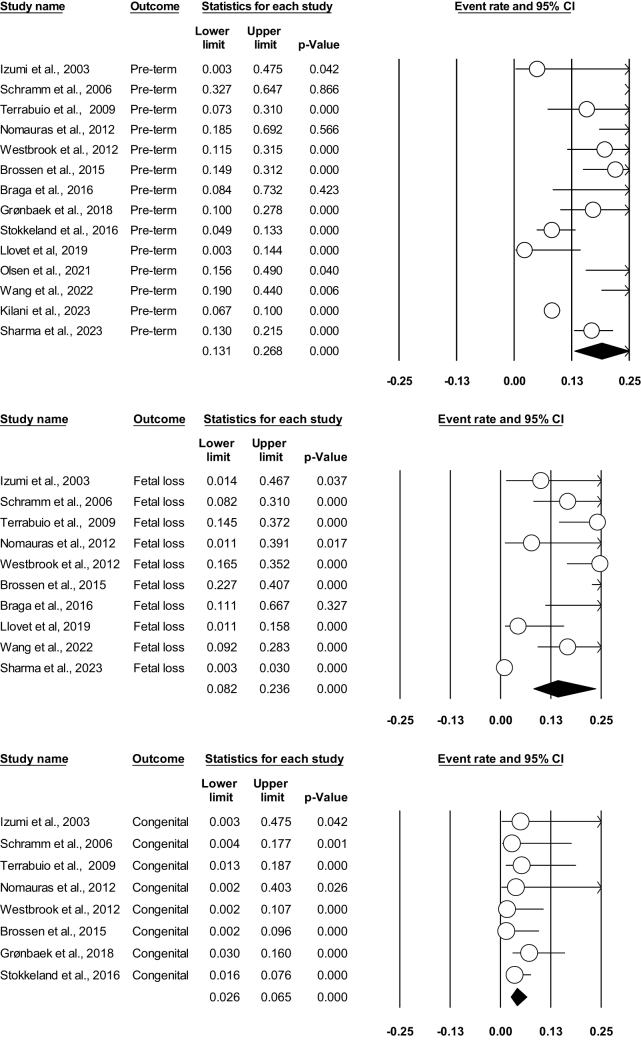
(A) Forest plot showing preterm births. (B) Forest plot showing fetal loss. (C) Forest plot showing congenital anomalies.

In our meta-analysis, 14 studies evaluated this outcome^[3,14,17–28]^. There was a statistically significant association in preterm birth and women with AIH (*E* = 0.190; 95% CI: 0.131–0.268; *P* = 0.000), and high evidence of heterogeneity was found among the included studies (*I*^2^ = 86.722%; *P* = 0.000). Figure 4A illustrates the forest plot for preterm birth.

##### Fetal loss

Ten studies were found to assess fetal demise (defined as pregnancy loss after viability threshold, i.e., 24 weeks) events^[3,14–18,20–22,24]^. In our analysis, we found that the results were significant (*E* = 0.142, 95% CI: 0.082–0.236; *P* = 0.000). On calculating heterogeneity via fixed effect mode, it was found to be high (*I*^2^ = 81.56%; *P* = 0.000) (Fig. 4B).

##### Congenital anomalies

Eight of our included studies reported congenital anomalies^[3,17–19,21,23,26,27]^. There was a statistically significant association between the outcome and AIH (*E* = 0.041, 95% CI: 0.026–0.065; *P* = 0.000), and no evidence of heterogeneity was found among the included studies (*I*^2^ = 0; *P* = 0.775) (Fig. 4C).

## Discussion

Pregnancy is associated with higher risk of maternal and fetal complications in people with AIH^[29]^. Pregnancy risks are determined by the severity of the condition, the presence of comorbidities, and the type of treatment used. An aggressive condition with liver cirrhosis and portal hypertension, or repeated flares, may pose additional dangers to the pregnant patient^[30]^.

It is crucial to describe disease context in terms of effects on both maternal and fetal outcomes; hence, we elaborated results as both outcomes. One of the maternal outcomes was a disease flare up. In general, during pregnancy, there is an increased tolerance capacity of the immune system to an increase in anti-inflammatory cells and cytokines^[29,31]^. This suggests that disease activity may improve during pregnancy, allowing for reduced drug dosage, while also undergoing flare in the postpartum period^[32–34]^. This lies in context with most of the studies included in our review where the disease underwent a significant flare up, post-partum. Olsen *et al*. reported that specifically AIH type 2 was associated with a trend toward a higher rate of postpartum flare (100% vs. 48%; *P* = 0.053)^[28]^. Llovet *et al*. also demonstrated that patients who had postpartum flares in their study showed an aggressive course, requiring stronger and combination therapies or even second-line therapy to achieve remission, suggesting a more immunologically active disease^[20]^. However, gestational flare up was reported as well in a few patients. Flare was the most reported complication reported in 33% of pregnancies (median AST 200 IU/L). Of them, 20 occurred during post-partum (within the first 3 months following delivery), while six occurred during gestation^[3]^.

The prognosis of AIH during pregnancy has been a matter of debate and aggressive research for years owing to its difficult predictability^[15]^. Literature holds multiple case series reporting disease flare of severe type, during stable pregnancies and patients with no complications like cirrhosis or portal hypertension^[35,36]^. Nonetheless, older people appear to have a higher likelihood of experiencing flares during pregnancy. Moreover, if there was a flare the year before conception or if the patient was not taking medication (e.g., prednisone) during pregnancy, particularly if azathioprine was stopped before conception, all contributed to a flare up. This disease activity may be the main contributing factor to maternal mortality in pregnancy following AIH. In our analysis, maternal mortality emerged as a noteworthy concern, with a statistically significant increase in mortality among women with AIH. Some common complications that led to death reported were variceal bleed and embolism^[18]^.

Preterm delivery is defined as birth before the 37th week of pregnancy^[37]^. In AIH patients, the most frequently reported obstetric complication is preterm delivery^[14,27,33]^. In previous literature, the incidence of spontaneous preterm delivery is in consistency with our result, that is, AIH increased the probability of having a preterm neonate. The pathophysiology behind this is still vague, but it is postulated that it is probably secondary to the pro-inflammatory state due to AIH^[24]^. These patients may also have a higher risk of fetal growth restriction or small for gestational age babies^[19]^. Severe cases were more likely to experience this obstetric complication, especially when portal hypertension or cirrhosis were present^[38]^.

Miscarriage occurs when an unviable fetus is lost during pregnancy. The existing data on AIH patients are conflicting, with some publications indicating a higher incidence of miscarriage^[18,39]^. This reinforces the findings in our analysis as well. However, another population-based study could not confirm prior findings, suggesting that miscarriage rates in AIH patients are equivalent to those in low-risk pregnancy groups^[19,27]^.

Congenital malformations demonstrate variable prevalence in literature^[38,40]^. While our analysis found a link between AIH and anomalies, the previous few did not report any. During a 6-month screening program for congenital abnormalities in Brazil, 3.5% of 1197 livebirths were screened, with AIH reports ranging from 0% to 5.7%^[33,38,40]^. Congenital anomalies may be attributed to an increase in use of immunosuppressants and corticosteroids for AIH rather than a complication of the disease itself. The use of corticosteroids seems safe during pregnancy, some recent studies reported an increase in the incidence of congenital malformations with the use of this type of medication^[41]^.

We recognize several strengths in our study compared to existing literature. Our meta-analysis provides a comprehensive synthesis of AIH-related pregnancy outcomes by incorporating data from multiple studies, offering a broad representation of maternal and fetal risks. Additionally, our study focuses on clinically relevant outcomes, such as disease flare-ups and mortality, ensuring its applicability in clinical practice. However, we acknowledge certain limitations when compared to other studies. Some previous research has included control groups (e.g., AIH vs. non-AIH pregnancies), enabling direct risk comparisons, whereas our study does not have a distinct comparator. Moreover, variability in study designs, patient populations, and methodologies across the 14 studies may have contributed to the heterogeneity. Additionally, the potential for publication bias, as illustrated in the publication bias analysis, may influence the overall interpretation of the results. Another limitation is the limited association between other comorbidities in the mother during pregnancy with AIH. Some outcomes, such as gestational diabetes and maternal mortality, relied on a limited number of studies, potentially compromising the robustness of our conclusions. Additionally, our study included women with cirrhosis, which may have contributed to worse maternal outcomes, including higher mortality rates. Cirrhosis is an established risk factor for obstetric complications, including hepatic decompensation, variceal bleeding, and increased maternal mortality. Given that a significant proportion of the included studies did not stratify outcomes based on the presence or absence of cirrhosis, we were unable to perform a separate subgroup analysis to isolate its independent effect on maternal mortality. Future studies should focus on differentiating AIH outcomes based on cirrhosis status to provide a clearer risk assessment. Our study also did not collect specific data on medication use, including corticosteroids such as prednisone. The omission was due to variations in reporting across included studies, as many did not provide detailed treatment regimens. Given that corticosteroid use has been linked to an increased risk of GDM, future research should investigate how immunosuppressive therapy influences metabolic and obstetric outcomes in AIH pregnancies. Standardized reporting of medication use in AIH studies will be crucial to understanding these associations. Lastly, while the meta-analysis provides valuable quantitative insights, the complexity of AIH and its interactions during pregnancy may not be fully captured. These limitations highlight the need for cautious interpretation and highlight areas for improvement in future research endeavors.

Nevertheless, the comprehensive synthesis of data in this systematic review sheds light on the intricate relationship between AIH and pregnancy, providing essential insights for clinical management. The multifaceted nature of AIH, particularly its potential impact during pregnancy, necessitates a thorough examination of various outcomes. Our study addresses this need, considering maternal and fetal perspectives, disease activity, and potential complications.

## Conclusion

The heightened risks identified, including increased disease flare, maternal mortality, GDM, preterm births, fetal loss, and congenital anomalies, highlight the importance of vigilant clinical management for pregnant individuals with AIH. The observed heterogeneity in some outcomes necessitates further investigation into the underlying factors influencing disease dynamics during pregnancy. Clinicians should consider these findings in the context of individual patient characteristics, emphasizing personalized care and tailored interventions.

## Data Availability

Data can be available upon reasonable request to the corresponding author.

## Research registration unique identifying number (UIN)

Registry used: PROSPERO. Unique identifying number or registration ID: CRD42024511790 (https://www.crd.york.ac.uk/PROSPERO/view/CRD42024511790).
